# Clinical and immunological characteristics of TGM3 in pan-cancer: A potential prognostic biomarker

**DOI:** 10.3389/fgene.2022.993438

**Published:** 2023-01-06

**Authors:** Wenqing Zhang, Chenglong Wu, Kaili Zhou, Yu Cao, Wange Zhou, Xue Zhang, Dan Deng

**Affiliations:** ^1^ Dermatology Center, Xinhua Hospital, Shanghai Jiaotong University School of Medicine, Shanghai, China; ^2^ Department of Dermatology, Xinhua Hospital, Shanghai Jiaotong University School of Medicine, Shanghai, China; ^3^ Institute of Dermatology, Shanghai Jiaotong University School of Medicine, Shanghai, China; ^4^ Department of Dermatology, Shanghai Children's Medical Center, Shanghai Jiaotong University School of Medicine, Shanghai, China

**Keywords:** TGM3, immunotherapy, immune response, prognosis, pan-cancer

## Abstract

**Background:** Recent studies have identified that transglutaminases (TGMs) are involved in a widespread epigenetic modification in tumorigenesis. However, it remains unclear how transglutaminase 3 (TGM3) affects in pan-cancer. The present study aimed to explore the clinical and prognostic function of TGM3 in pan-cancer as well as to explore the relationship of TGM3 expression with clinical stage, survival rate, prognosis condition, immune infiltration and mutation indicators.

**Methods:** The relevant data of tumors were obtained from The Cancer Genome Atlas (TCGA), TARGET, Cancer Cell Line Encyclopedia (CCLE) and Genotype-Tissue Expression (GTEx) databases. According to the Human Protein Atlas (HPA) and TIMER databases, we evaluated the protein expression levels of TGM3 in different organs and tissues as well as their association with immune cell infiltration and immunotherapeutic response in pan-cancers. Expression differences between normal and tumor tissues as well as survival and prognosis situation, clinical data characteristics, tumor mutational burden (TMB), microsatellite instability (MSI), and RNA methylation were also assessed. Oncogenic analyses were also evaluated by GSEA.

**Results:** Compared to normal tissues, some tumor tissues had a lower expression level of TGM3, while other tumor tissues had a high expression level of TGM3. Further studies showed that high TGM3 expression had a certain risk impact on pan-cancer as high TGM3 expression levels were detrimental to the survival of several cancers, except for pancreatic cancer (PAAD). High expression level of TGM3 was also related to higher clinical stages in most cancers. The expression level of TGM3 was significantly negatively correlated with the expression of immune infiltration-related cells, including B cells, CD8+ T cells, CD4+ T cells, neutrophils, macrophages and dendritic cells (DCs). Furthermore, in most cancer types, TGM3 was inversely correlated with TMB, MSI, and methylation, suggesting that TGM3 expression can be used to assess potential therapeutic response, especially immune-related targeted therapy. GSEA analysis elucidated the biological and molecular function of TGM3 in various cancer types. Taken together, these bioinformatic analyses identified TGM3 as an important biomarker for clinical tumor prognosis and evaluation of treatment efficacy.

**Conclusion:** We comprehensively analyzed the clinical characteristics, tumor stages, immune infiltration, methylation level, gene mutation, functional enrichment analysis and immunotherapeutic value of TGM3 in pan-cancer, providing implications for the function of TGM3 and its role in clinical treatment.

## Introduction

Transglutaminases (TGMs) are engaged in a number of physiologically important protein cross-linking reactions and promote the formation of rigid macromolecular complexes (Lorand and Graham, 2003). By introducing isopeptide bonds between lysine and glutamine residues in target proteins, these enzymes catalyze the construction of protein networks that promote protective barriers in the normal epidermis and microenvironment against tumorigenesis. TGMs are involved in several post-histone modifications including deamidation of glutamine residues, covalent binding of polyamines and esterification of lipids, help form solid and insoluble protein complexes for hair fibers, hair follicles and the epidermis (Lorand and Conrad, 1984; Nemes et al., 1999; Chermnykh et al., 2020). Transglutaminase 3 (TGM3) is a protein responsible for the process of epidermal formation and belongs to the cross-linking enzyme family, which has been discovered in hair fibers, hair follicles and the epidermis, as well as in other organs, including mucosa, brain, stomach, spleen, small intestine, testis, and skeletal muscle (Buxman and Wuepper, 1976; Ogawa and Goldsmith, 1976; Kim et al., 1999; Hitomi et al., 2003; Zhang et al., 2005). In addition to its constitutive expression, studies have primarily reported that TGM3 is a tumor-related suppressor in carcinogenesis that is involved in the apoptosis mechanism (Wu et al., 2013; Wu et al., 2018; Feng et al., 2020).

As an epithelial-derived molecule, TGM3 may play a key role in the tumorigenesis of various epithelial cancers (Chermnykh et al., 2020). TGM3 is either down- or upregulated in various cancers, including oral cancer, laryngeal cancer, esophageal cancer, colorectal cancer and hepatocellular cancer (Chen et al., 2000; Mendez et al., 2007; Negishi et al., 2010; Liu et al., 2012; Nayak et al., 2019; Smirnov et al., 2019; Hu et al., 2020). TGM3 expression is correlated with cellular dedifferentiation, cellular proliferation, increased invasiveness, lymph node metastasis and hematogenous recurrence, which results in high recurrence rate, rapid progression and unfavorable prognosis (Smirnov et al., 2019). Studies have shown that loss of DNA heterozygous mutations and elevated CpG island methylation in the TGM3 promoter, which in turn silenced transcription and down-regulated TGM3 expression in laryngeal carcinomas (He et al., 2002). Additionally, other studies have revealed that ectopic expression of TGM3 decreases the protein expression level of full-length PARP, procaspase-3, procaspase-8 and Bcl-2 but increase the protein expression of cleaved PARP and Bax protein in head and neck squamous cell carcinomas cell lines (Wu et al., 2013). TGM3 is essential in the proliferation of epithelial cell carcinomas by regulating epithelial-to-mesenchymal transition (EMT) *via* hindering the PI3K/AKT signaling pathway, indicating that TGM3 may be a potential therapeutic target for prevention of tumorigenesis and metastasis (§ et al., 2000; Xu et al., 2014).

We performed a bioinformatics analysis of TGM3-related bioinformatics data to assess the expression level of TGM3 in different tissues and its correlation with different types of cancers. Using an online database, we found that TGM3 was differently expressed in numerous cancer types. The expression level was closely correlated to survival, immune cell infiltration and tumor mutation status. Additionally, TGM3 also influenced methylation modification in some cancer types. These results identified that TGM3 may serve as a marker of malignancy risk and clinical prognosis as well as an indicator of immunotherapy response and a potential target for cancer therapy, especially in some poorly curative or highly drug-resistant tumors.

## Materials and methods

### Data collection and processing

The gene expression data and clinicopathological and prognostic information of 34 human cancers in TCGA, TARGET and Genotype-Tissue Expression (GTEx) were downloaded from the portal website for analysis (https://xenabrowser.net/). Cancer Cell Line Encyclopedia (CCLE) database were extracted through the portal website for cell line analysis. The entire data collection was filtered and transformed by log2 (X + 0.001) or log2 (TPM + 1), using the RMA package in R (R studio version: 1.2.1335, R version: 3.6.4) (Team, 2015a; Team, 2015b). Clinical information, such as age, sex and clinical stages was also downloaded from the related websites. GEPIA2 (Gene Expression Profiling Interactive Analysis) (http://gepia2.cancer-pku.cn/) is a useful web-served tool based on TCGA and GTEx database to reveal the relationship between TGM3 expression level and tumor clinical stages through the stage plot package in R (Tang et al., 2019). Further, the downloaded information from the databases was utilized to analyze tumor mutational burden (TMB), microsatellite instability (MSI), mis-match repair (MMR), and DNA methylation. TMB refers to the number of somatic mutations per megabase in the whole genome of cancer samples, excluding germline DNA variants, which acts as an indicator to reflect the total degree of gene mutation in tumor cells. Tumors with high levels of TMB represent high levels of gene mutations in their tumor cells, reflecting more tumor neoantigens recognized by the immune system. MSI refers to the phenomenon in which new microsatellite alleles appeared at a microsatellite locus at a certain genomic location in tumors due to any change in repeat unit length. Moreover, the protein expression level of TGM3 in pathological tissue of various tumors was obtained from the HPA database (www.proteinatlas.org/pathology, accessed in April 2022).

### Analysis of survival prognosis

Cox regression analysis was performed to examine the correlation of TGM3 expression with overall survival (OS), disease-specific survival (DSS), disease-free interval (DFI) and progression-free interval (PFI) in each cancer type from the databases using the coxph function in the survival package in R (Survival, version 3.2–7) (rdocumentation.org/packages/survival). According to the optimal separation method in the maxstat package in R, patients were grouped into high and low TGM3 expression groups. Based on the survfit function in the survival package in R, Kaplan–Meier survival curves were constructed for each cancer type. The log-rank test was used to determine the specificity and time-dependent sensitivity of survival by deploying the survivalROC package in R and examine the difference between curves, in which *p* < 0.05 was considered significant (Team, 2015b).

### Investigation of immune-related factors

The relationship between TGM3 expression and immune infiltration was analyzed through the TIMER database (http://cistrome.org/TIMER/) (Li et al., 2016a). TIMER is database-derived webtool for the systematic analysis of immune infiltration scores of six common types of immune cells across diverse cancer types (Li et al., 2016a; Li et al., 2017). CIBERSORT, a deconvolution algorithm, was used to evaluate the relative number of immune cells of 22 different tumor-infiltrating lymphocyte subsets (Newman et al., 2015). We collected information on immunomodulators, including major histocompatibility complexes (MHCs), receptors, chemokines, immunostimulators and immunoinhibitors, from the study by Charoentong et al. (2017) and evaluated the correlation between TGM3 and these immune checkpoints and regulators. Based on the cutoff values obtained using X-tile, the patients were divided into two groups as follows: low-TGM3 expression group and high-TGM3 expression group. The Tumor Immune Dysfunction and Rejection (TIDE) algorithm was used for prediction of response to immune checkpoint inhibitor (ICI) treatment (Jiang et al., 2018).

### DNA methylation and mis-match repair analysis

The potential relevance of TGM3 expression with DNA methylation, MMR and RNA modifications in different pan-cancer tumors was investigated by downloading information from TCGA, TARGET, and GTEx databases. MMR genes (i.e., MLH1, MSH2, MSH6, PMS2 and EPCAM) played a crucial role in DNA replication error repair (Meiser et al., 2020). Aberrant DNA methylation is an inchoate incidence in tumorigenesis and development, and it may be related to valuable cancer biomarkers (Wu et al., 2021). RNA modification (i.e., m1A, m5C, and m6A) plays a vital role in a myriad of biological processes and cellular functions, including cell processing, nuclear export, translation, and even decay (Zhang et al., 2020).

### Biological role of transglutaminase 3 expression in tumors

Gene Set Enrichment Analysis (GSEA) was used to explore the biological functions of TGM3 in various tumors. Oncogenic signature genes and Kyoto Encyclopedia of Genes and Genomes (KEGG) gene sets were also obtained from the authorized portal website. The “c6.all.v7.4.symbols.gmt” and “c2.cp.kegg.v7.4.symbols.gmt” subsets were downloaded from the Molecular Signatures Database (MSigDB) to evaluate oncogenic or tumor-related pathways in pan-cancer, and functional analysis was performed using the limma, clusterProfiler, and enrichplot packages in R. *p* < 0.05 and FDR < 0.25 were considered statistically significant.

### Statistical analyses

In the present study, R and corresponding R packages were used to analyze the datasets from portal official websites. The results are presented as the mean ± standard deviation (SD). *p* < 0.05 in two-tailed tests was regarded as statistically significant. Continuous variables were assessed by Student’s t-test with unpaired samples and by a paired *t*-test with paired samples. Spearman’s correlation test was used to explain the relevance between TGM3 expression and several parameters, such as immune infiltration scores of six common types of immune cells, TMB, MSI, MMR genes and methylation transferase genes. *p* < 0.05 was considered statistically significant. The ggplot2 and forestplot packages in R were used to draw the relevant graphs (Ginestet, 2011).

## Results

### Transglutaminase 3 expression analysis in pan-cancer

**FIGURE 1:**
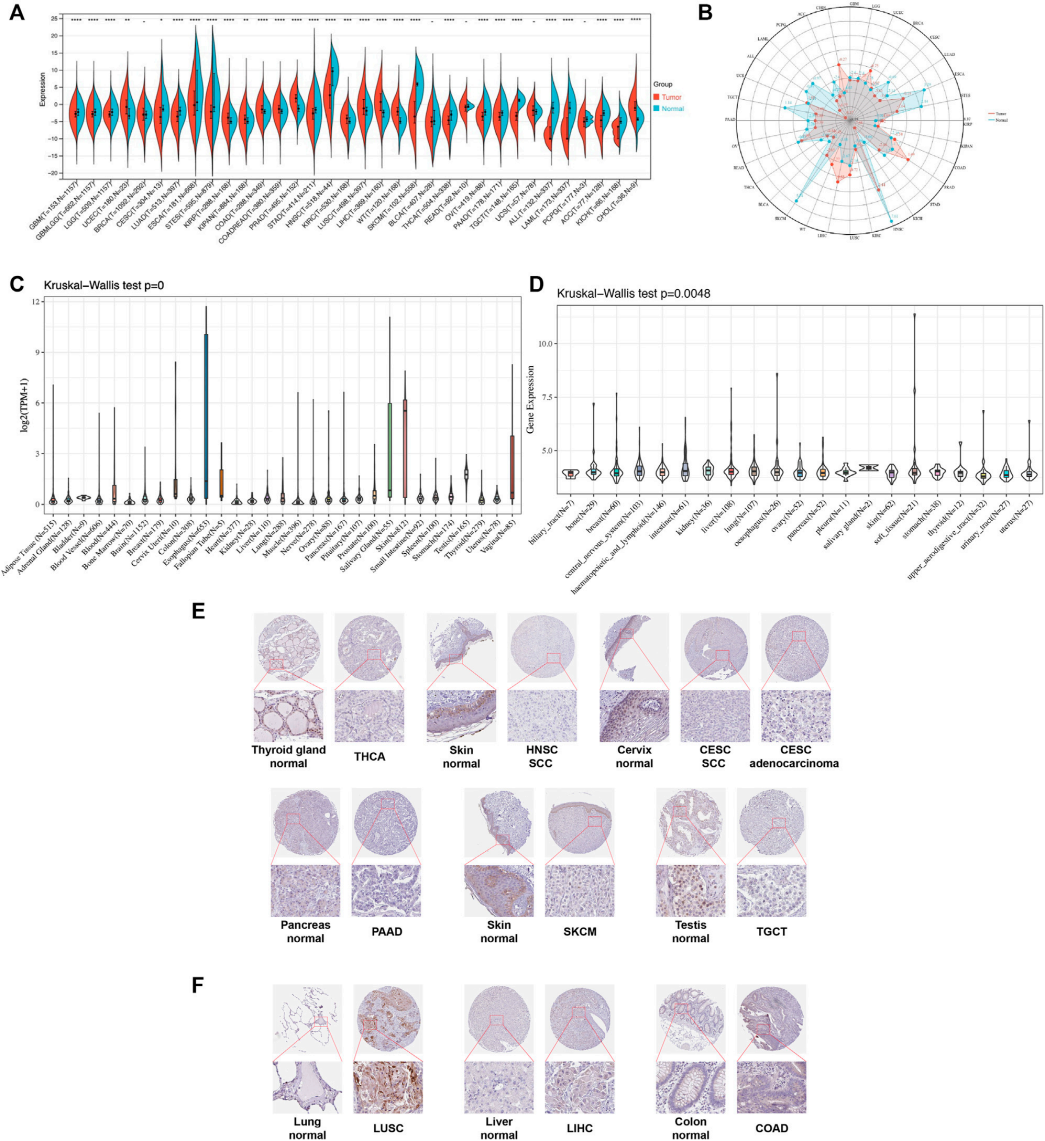
Pan-cancer TGM3 expression. **(A)** Pan-cancer expression of TGM3 between tumor tissues and normal tissues from TCGA, TARGET, and GTEx databases. *p < 0.05, **p < 0.01, ***p < 0.001 and ****p < 0.0001. **(B)** Expression levels of TGM3 in tumor types from the TCGA database. Dots represent the mean of TGM3 expression in each tumor type. **(C)** Normal mRNA expression levels of TGM3 in different tissues from the GTEx database. **(D)** mRNA expression levels of TGM3 in different tumor cell lineages obtained from the CCLE database. **(E)** Lower expression of TGM3 at the protein level in tumor tissues of THCA, HNSC, CESC, PAAD, SKCM, and TGCT by IHC in HPA database (X50, X400). **(F)** Higher expression of TGM3 at the protein level in tumor tissues of LUSC, LIHC and COAD by IHC in HPA database (X50, X400).

We first evaluated the expression level of TGM3 in TCGA, TARGET and GTEx databases of pan-cancer. Compared to the respective normal groups, TGM3 expression was higher in the cancer groups, including uterine corpus endometrial carcinoma (UCEC), kidney renal papillary cell carcinoma (KIRP), pan-kidney cohort (KIPAN), colon adenocarcinoma (COAD), prostate adenocarcinoma (PRAD), colon and rectum adenocarcinoma (COADREAD), kidney renal clear cell carcinoma (KIRC), lung squamous cell carcinoma (LUSC), liver hepatocellular carcinoma (LIHC), high-risk Wilms tumor (WT) and cholangiocarcinoma (CHOL). Moreover, a lower expression of TGM3 was found in glioblastoma multiforme (GBM), brain lower grade glioma (LGG), cervical squamous cell carcinoma and endocervical adenocarcinoma (CESC), lung adenocarcinoma (LUAD), esophageal carcinoma (ESCA), stomach and esophageal carcinoma (STES), stomach adenocarcinoma (STAD), head and neck squamous cell carcinoma (HNSC), skin cutaneous melanoma (SKCM), glioma (GBMLGG), thyroid carcinoma (THCA), ovarian serous cystadenocarcinoma (OV), pancreatic adenocarcinoma (PAAD), testicular germ cell tumor (TGCT), acute lymphoblastic leukemia (ALL), acute myeloid leukemia (LAML), adrenocortical carcinoma (ACC) and kidney chromophobe (KICH) (Figures 1A,B).

Analysis of the data from GTEx, which was comprised of different normal tissue samples indicated that the mRNA expression levels of TGM3 were similar across all tissues with greater heterogeneity in the esophagus, salivary gland, vagina and skin (Figure 1C). Compared to the TGM3 expression levels in cell lines from the CCLE database, the TGM3 expression levels were slightly elevated with a narrow range of expression (Figure 1D).

To further evaluate TGM3 expression in human tissues, we examined TGM3 antibody staining in several tissues from the Human Protein Atlas (HPA) database (Hu et al., 2021). Six types of tumor tissues (THCA, HNSC, CESC, PAAD, SKCM and TGCT) were stained with lower TGM3 expression clusters compared with normal tissues (Figure 1E). Meanwhile, TGM3 IHC staining levels of LUSC, LIHC and COAD were little stronger than those in normal tissues (Figure 1F).

### Prognostic significance of transglutaminase 3

**FIGURE 2:**
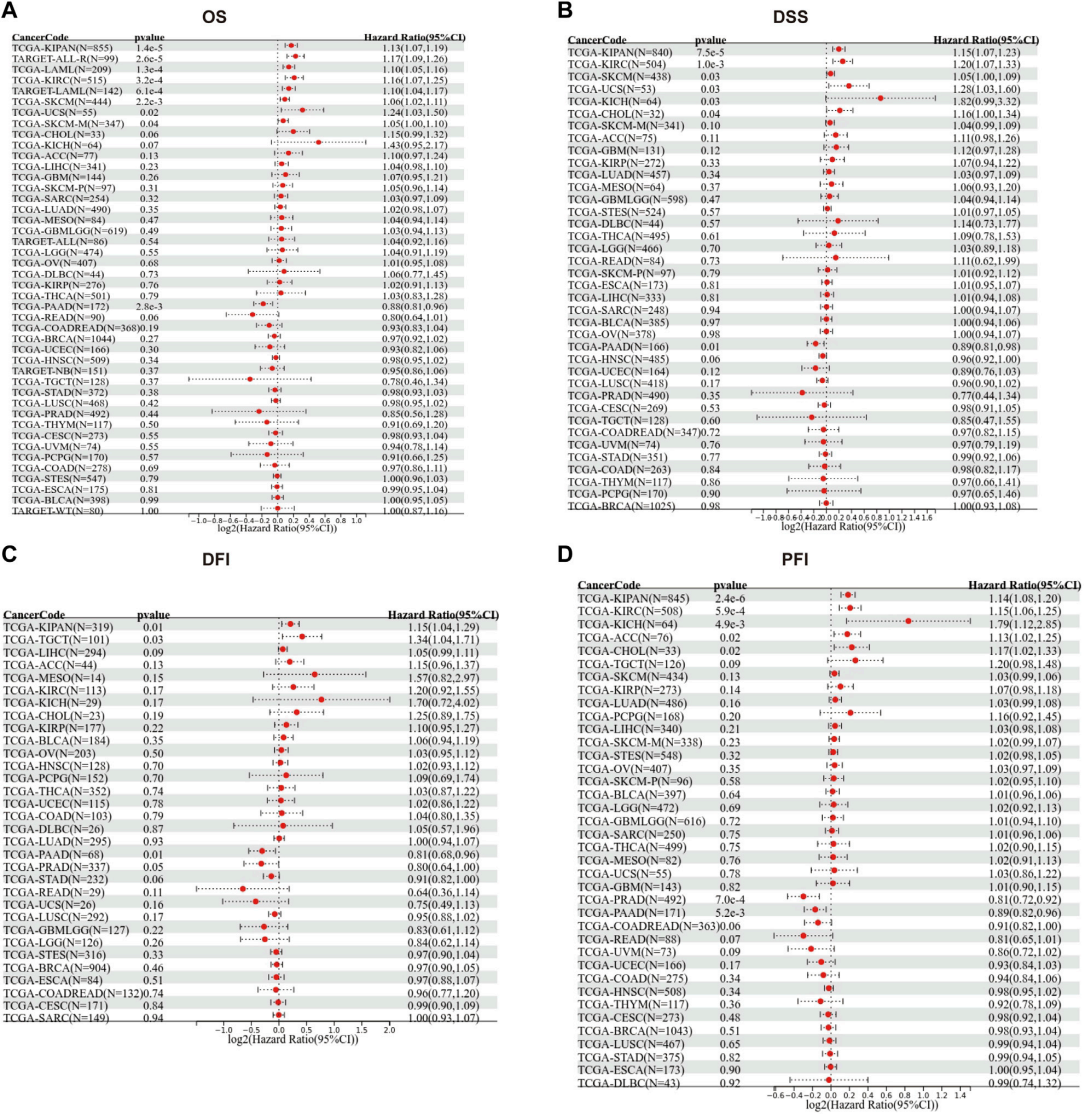
Forest maps showing the univariate Cox regression results of TGM3 for OS **(A)**, DSS **(B)**, DFI **(C)** and PFI **(D)** in pan-cancer analysis.

**FIGURE 3:**
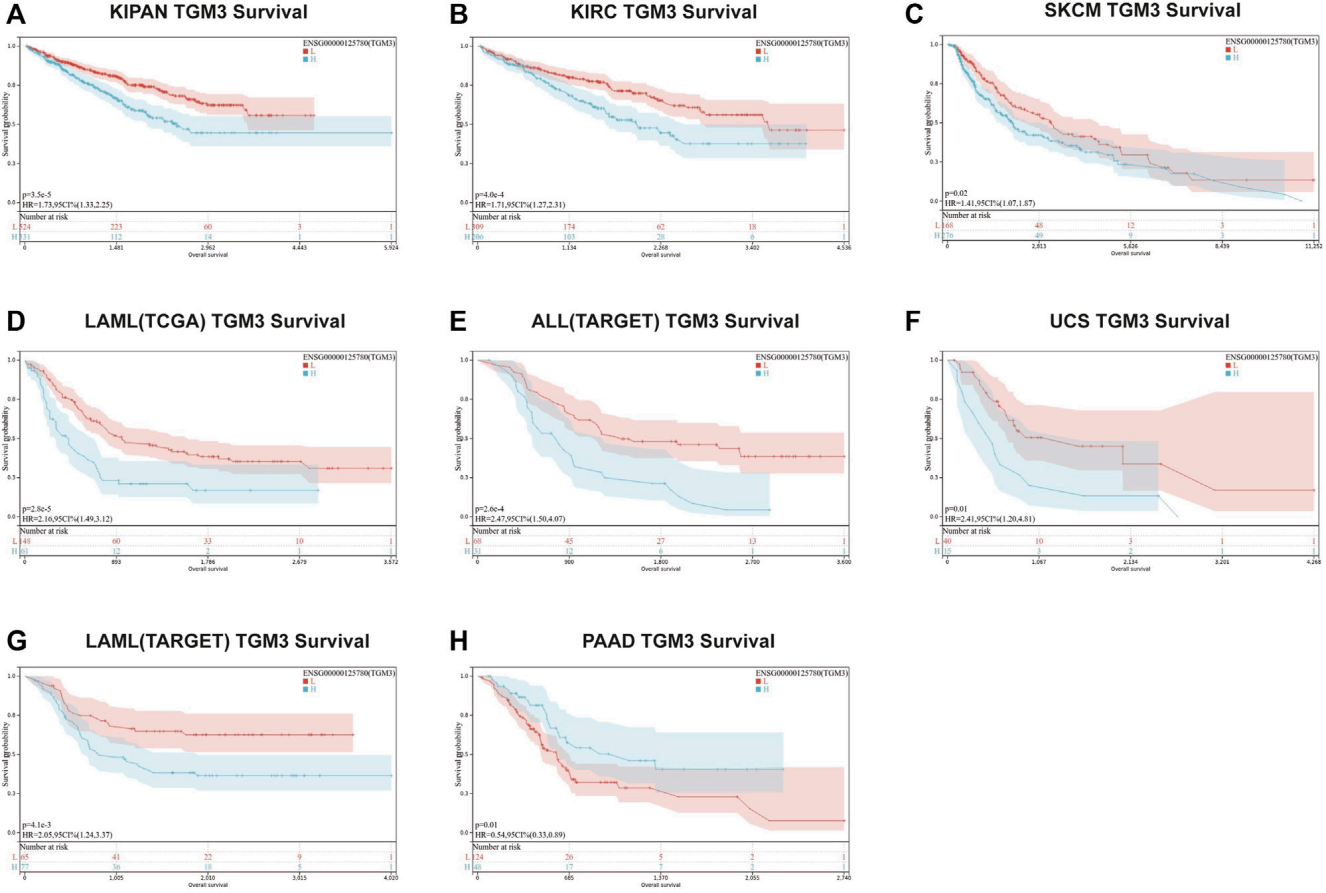
Kaplan–Meier OS analysis of TGM3. **(A–H)** Kaplan–Meier OS analysis of TGM3 in pan-cancer tumor types in TCGA and TARGET databases. The median value of TGM3 in each tumor was considered as the cutoff value.

We then evaluated the multifaceted prognostic value of TGM3 in cancers with univariate Cox regression analysis. Notably, TGM3 expression was significantly associated with OS in several cancer types. The hazard ratios for TGM3 were significant for KIPAN, KIRC, LAML, SKCM, uterine carcinosarcoma (UCS), ALL and PAAD, among which TGM3 had the highest risk effect in UCS and was a protective factor in PAAD (Figure 2A). DSS analysis revealed that TGM3 acted as a risk factor for KIPAN, KIRC, SKCM, UCS, KICH, and CHOL but a protective factor for PAAD (Figure 2B). DFI analysis revealed that TGM3 acted as a risk factor for KIPAN and TGCT but a protective factor for PAAD and PRAD (Figure 2C). Finally, PFI analysis showed that TGM3 acted as a risk factor for KIPAN, KIRC, ACC, KICH, and CHOL but a protective factor for PAAD and PRAD, which indicated that TGM3 may play a protective role in PAAD and PRAD with regard to prognosis (Figure 2D). Moreover, cut-off value was designed in each cancer type to construct an optimal model for further survival analysis. Kaplan–Meier OS analysis indicated that survival differences were significant in KIPAN, KIRC, SKCM, LAML, PAAD, UCS, and ALL (Figures 3A–H) (Supplementary Table S1). High expression level of TGM3 was detrimental to the survival in most cancer types, except PAAD (Figure 3H), which suggested that TGM3 may be a risk prognostic factor in most cancers.

### Clinical characteristics of transglutaminase 3 in pan-cancer

**FIGURE 4:**
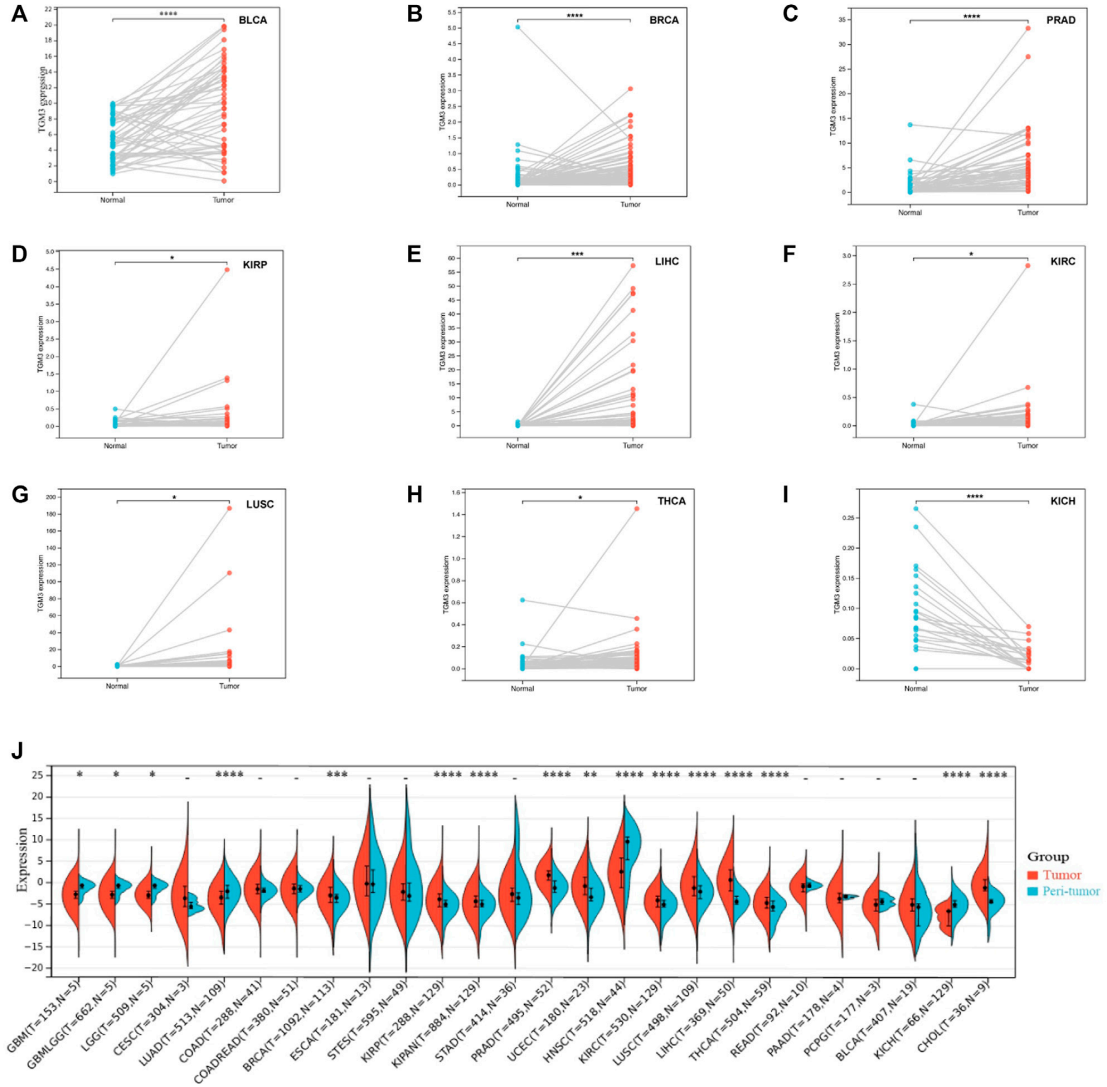
Paired TGM3 expression level in pan-cancer. **(A–I)** The differential expression of TGM3 in tumor lesions and adjacent normal tissues from TCGA database was examined. **(J)** mRNA expression differences of TGM3 between peri-tumor and tumor samples, integrating statistics from TCGA database. *p < 0.05, **p < 0.01, ***p < 0.001, and ****p < 0.0001.

We then analyzed the expression of TGM3 in different clinical stages according to the clinical tumor stage classification form TCGA pan-cancer. In some cancers, the expression level of TGM3 was associated with some clinical stages of cancers with slight statistical significance in KIPAN, KIRC, LIHC, OV, UVM and UCS (Supplementary Figure S1; Supplementary Table S2). Regarding paired tumor lesions and normal tissues samples in the pan-cancer database, TGM3 was expressed at high levels in bladder urothelial carcinoma (BLCA), breast invasive carcinoma (BRCA), PRAD, KIRP, LIHC, KIRC and LUSC (Figures 4A–G), but it was expressed at low levels in THCA and KICH (Figures 4H,I). Finally, the mRNA expression differences of TGM3 among peri-tumor and tumor samples were summarized by integrating statistics from TCGA databases (Figure 4J).

### Correlation of transglutaminase 3 expression with immune checkpoint and regulator genes

**FIGURE 5:**
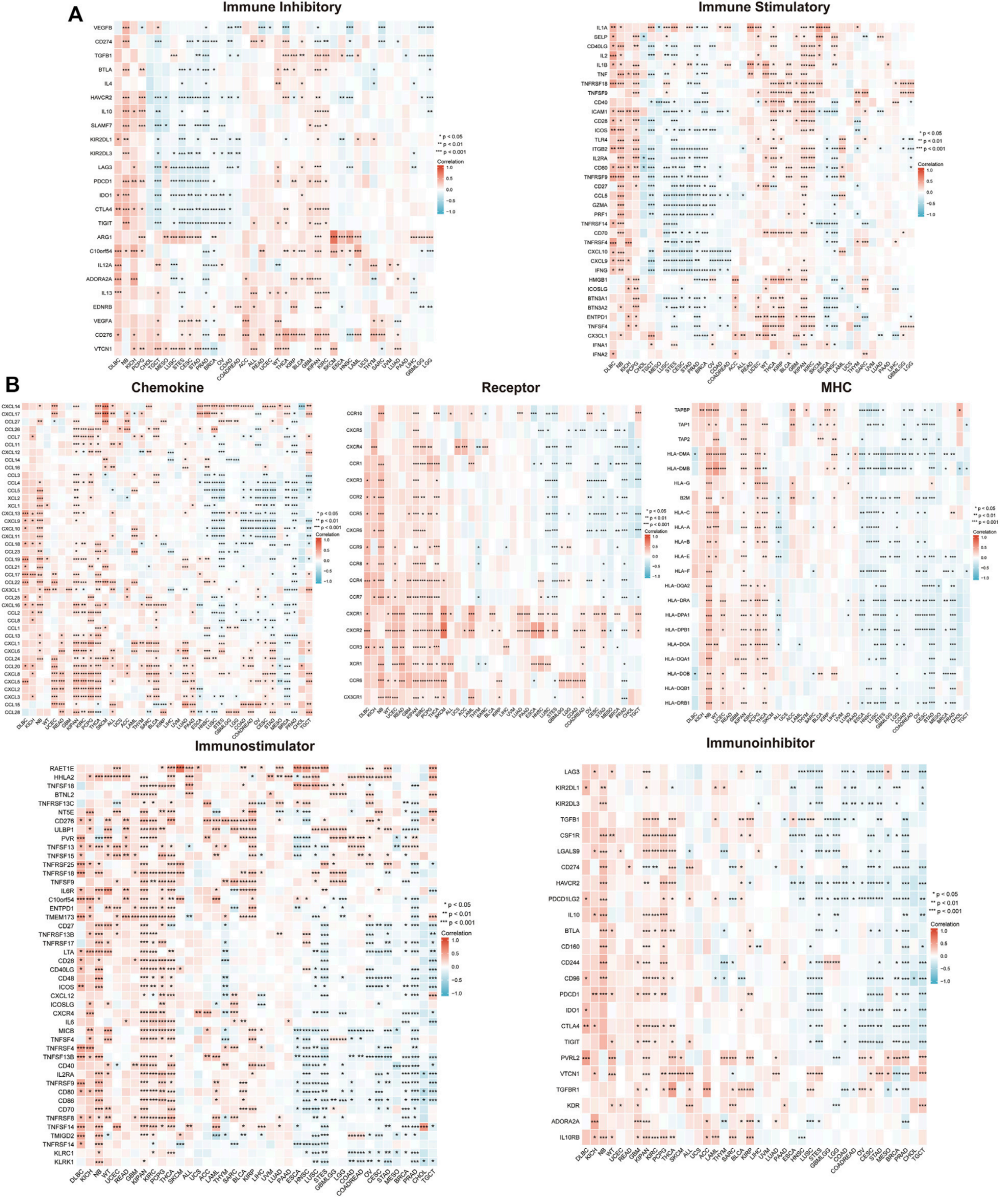
Correlation of TGM3 mRNA expression levels with immune checkpoint **(A)** and immune regulator **(B)** mRNA expression in pan-cancer from TCGA database. *p < 0.05, **p < 0.01 and ***p < 0.001.

The association of TGM3 with immune checkpoint genes and immune regulator genes was next assessed. We found a high correlation (*p* < 0.05) between TGM3 and immune checkpoint gene expression in several cancer types. The results implicated TGM3 in the regulation of the tumor immune response through negative modulation of immune inhibitory checkpoints, particularly for STES, LUSC, CESC, TGCT and PRAD, but without statistical significance. Moreover, the expression level of TGM3 was positively related with most immune checkpoint molecules in KICH, WT, THCA, PCPG, KIPAN, KIRC, DLBC and NB. In addition, there was a sharper contrast in immune stimulatory checkpoints, exhibiting TGM3 positive relations with DLBC, NB, KICH, PCPG, WT, THCA, KIRP, KIPAN and KIRC but negative relations with TGCT, LUSC, STES, CESC, STAD, PRAD and BRCA (Figure 5A).

Several genes acting as immune regulator components correlated with the tumor microenvironment (TME) in the immune response. TGM3 was closely correlated with immune regulator gene expression and had a high correlation (*p* < 0.05) with chemokine receptors, MHCs, immunoinhibitors, and immunostimulators in various cancer types. Furthermore, significant active coexpression of TGM3 with immune regulator genes was detected in PCPG, THCA, KIPAN, KICH, KIRC and neuroblastoma (NB). In PRAD, STES, CESC, LUSC and TGCT, however, TGM3 partly negatively regulated the expression level of immune regulator genes, including MHC, immunoinhibitor, and immunostimulator genes (Figure 5B).

### Correlation of transglutaminase 3 expression with tumor-infiltrating immune cells

**FIGURE 6:**
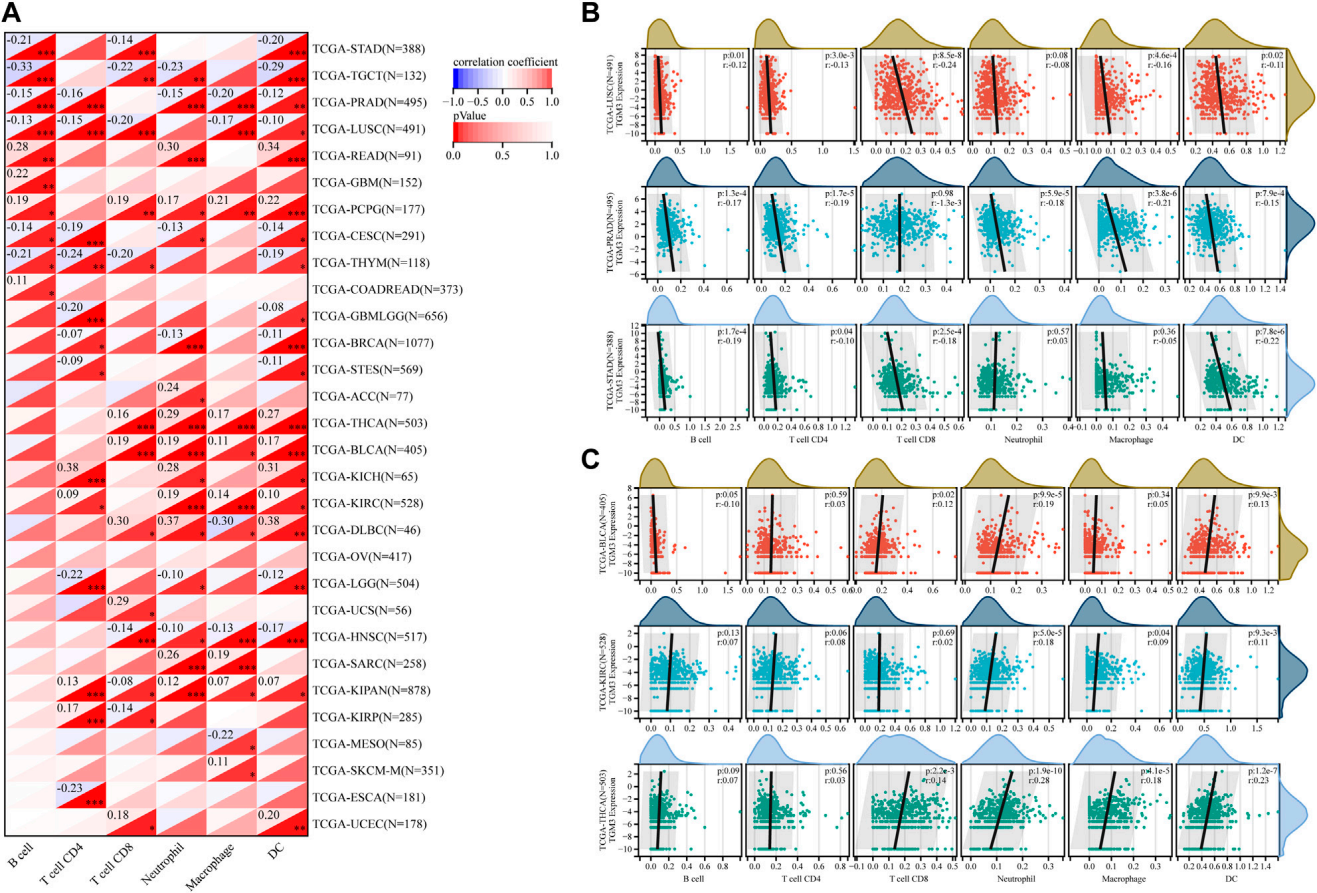
**(A)** Based on the TIMER database, the correlation between TGM3 expression and tumor-infiltrating immune cells was demonstrated. **(B)** Negative correlation between TGM3 mRNA expression and six immune cell infiltration scores in LUSC, PRAD and STAD. **(C)** Positive correlation between TGM3 mRNA expression and six immune cell infiltration scores in BLCA, KIRC and THCA. *p < 0.05, **p < 0.01, and ***p < 0.001.

The TIMER database was then utilized to evaluate the relationship between TGM3 expression and tumor-infiltrating immune cells, which revealed closely association with the immune infiltration of B cells, CD4+ cells, CD8+ cells, neutrophils, macrophages and DCs (Figure 6A).

Because the data suggested that TGM3 may affect immune cell differentiation, we assessed the relationship between TGM3 expression and the level of infiltrating immune cells in each cancer type from pan-cancer to elucidate how TGM3 regulates the immune microenvironment of tumors. The three top-ranking tumor cohorts of negative correlation were LUSC, PRAD and STAD (Figure 6B), which indicated that high TGM3 expression was associated with a potential decreased immune cell infiltration level. Of note, among all cell types in these three cancers, B cells, DCs, and neutrophils had higher coefficients compared to the other cell types. The three top-ranking tumor cohorts of positive correlation were BLCA, KIRC and THCA, in which macrophages, DCs and neutrophils had higher significant coefficients (Figure 6C).

### Correlation of transglutaminase 3 with tumor mutational burden, microsatellite instability and RNA methylation in cancers

**FIGURE 7:**
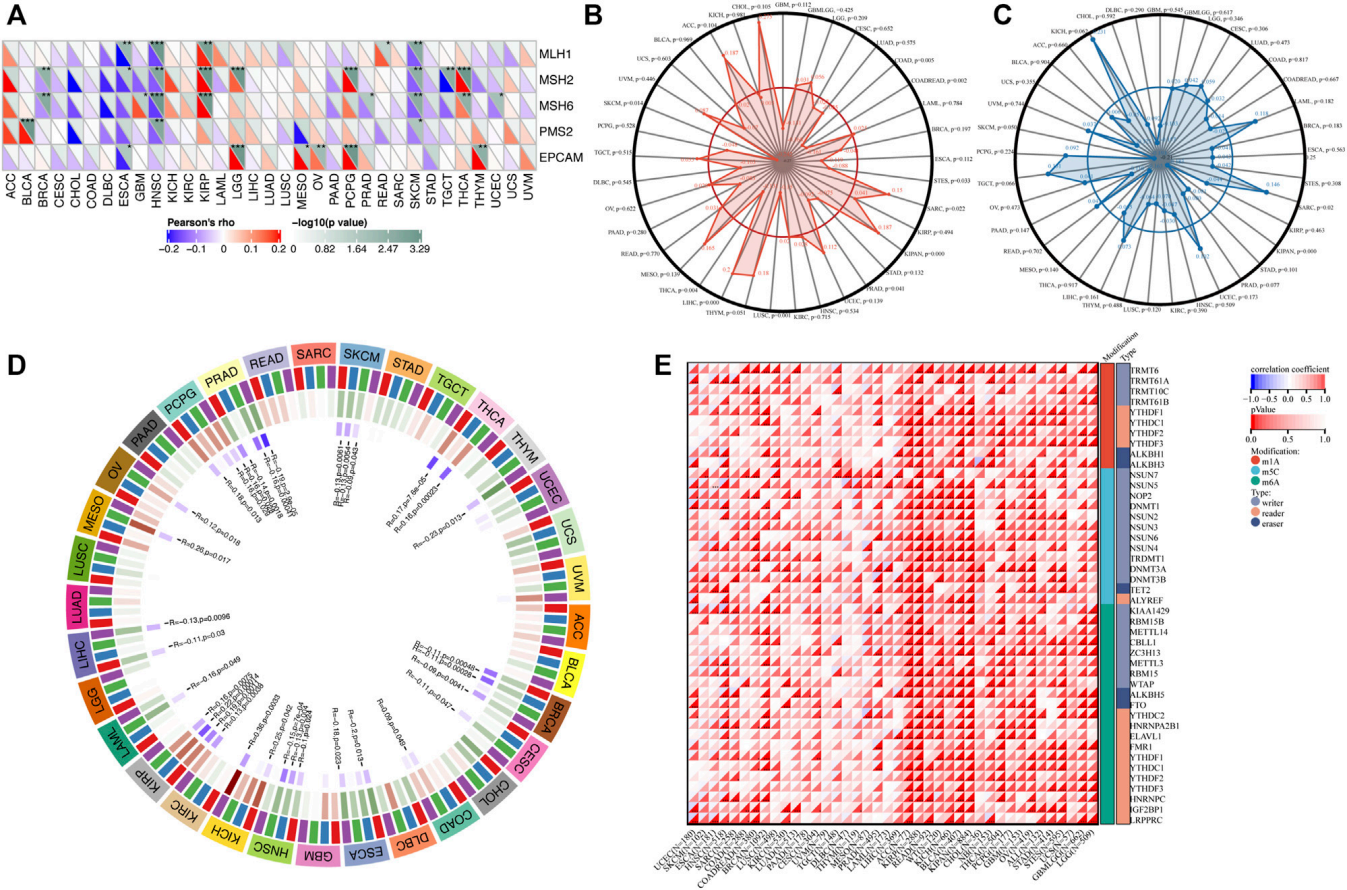
**(A)** Correlation between TGM3 mRNA expression and the expression of MMR genes, including MLH1, MSH2, MSH6, PMS2, and EPCAM. *p < 0.05, **p < 0.01, and ***p < 0.001 **(B)** Correlation between TMB and TGM3 expression. **(C)** Correlation between MSI and TGM3 expression. (*p* < 0.05 was considered significant). **(D)** Correlation between the mRNA expression level of TGM3 and those of four methyltransferases (DNMT1, red; DNMT2, blue; DNMT3A, green; and DNMT3B, purple). **(E)** Correlation between TGM3 and genes related to three types of RNA modifications (m1A, m5C and m6A). The sloping upper triangle in each figure indicates the coefficients calculated by Spearman’s correlation test, and the sloping lower triangle indicates the *p*-value. *p < 0.05, **p < 0.01, and ***p < 0.001.

We next investigated the relationship between TGM3 expression and somatic copy number alteration signatures to evaluate the clinical effects of ICIs in tumors with low and high expression level of TGM3. Regarding the relationship between TGM3 and MMR genes, TGM3 was closely related to MLH1, MSH2, MSH6, PMS2 and EPCAM in HNSC, KIRP and SKCM (Figure 7A). Analysis of the relationship between TGM3 gene expression level and TMB (Supplementary Figure S2) demonstrated that TGM3 negatively regulated TMB in COAD, COADREAD, STES, PRAD, LUSC, THCA and SKCM (R < 0, *p* < 0.05) but positively regulated TMB in SARC, KIPAN, and LIHC (R > 0, *p* < 0.05) (Figure 7B). Spearman’s correlation coefficient was also utilized to explain the correlation with TGM3 gene expression (Supplementary Figure S3), which demonstrated that TGM3 was negatively correlated with MSI in KIPAN but positively correlated with MSI in SARC (Figure 7C). Regarding the association between TGM3 expression level and that of four methylation transferases (DNMT1, DNMT2, DNMT3A, and DNMT3B), coexpression of these genes was found in PCPG, PRAD, SKCM, THCA, THYM, BRCA, CESC, COAD, ESCA, HNSC, KICH, KIRC, KIRP, LAML, LGG, LIHC, and OV. Of note, KIRP and PRAD demonstrated significantly high coexpression coefficients (Figure 7D).

We next investigated the associations between TGM3 expression and tumorigenesis mechanisms. In particular, we evaluated the relationship of TGM3 expression with genes related to three types of RNA modifications, namely, m1A (10 genes), m5C (13 genes) and m6A (21genes), using an online database. In most cancers, TGM3 expression levels were positively correlated with most m1A-, m5C- and m6A-modified genes, especially in high-risk Wilms tumor (WT), GBM, OV, KIRP, KIPAN, ACC, READ, SARC, BRCA, LGG, GBMLGG, THCA and KIRC (Figure 7E).

**FIGURE 8:**
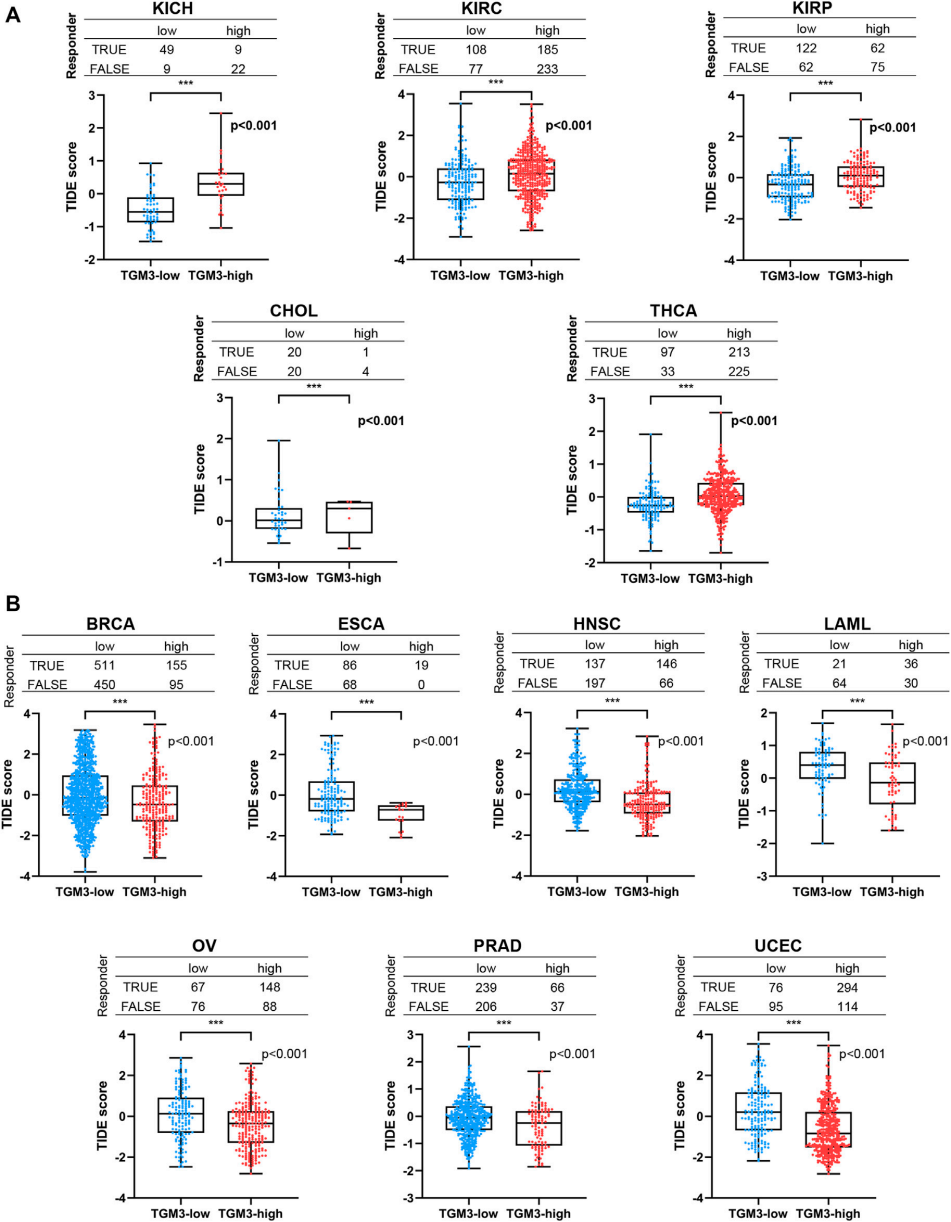
The relationship between TGM3 and patient response to ICI therapy. **(A)** The TGM3-low tumor group had a better therapeutic ICI response in KICH, KIRC, KIRP, CHOL and THCA. **(B)** The TGM3-high tumor group had a better therapeutic ICI response in BRCA, ESCA, HNSC, LAML, OV, PRAD and UCEC.

Furthermore, TIDE analysis was provided to evaluate if TGM3 has the potential therapeutic target for ICI therapy. We separated the cancers into low-TGM3 expression and high-TGM3 expression tumor groups. The group with lower TGM3 responded better to ICI therapy in KICH, KIRC, KIRP, CHOL and THCA with statistically considerable differences, which showed that lower TGM3 levels would increase the positive correlation between cytotoxic T lymphocytes and survival benefits (Figure 8A). While the group with higher TGM3 expression of tumors responded better to ICI therapy in BRCA, ESCA, HNSC, LAML, OV, PRAD and UCEC with statistically considerable differences (Figure 8B). These results implied the role of TGM3 in regulating tumor immune escape and immunotherapeutic resistance in different cancers. Other cancer types responded better to ICI therapy were exhibited in Supplementary Figure S4.

### Oncogenic analysis of transglutaminase 3 expression by gene set enrichment analysis

**FIGURE 9:**
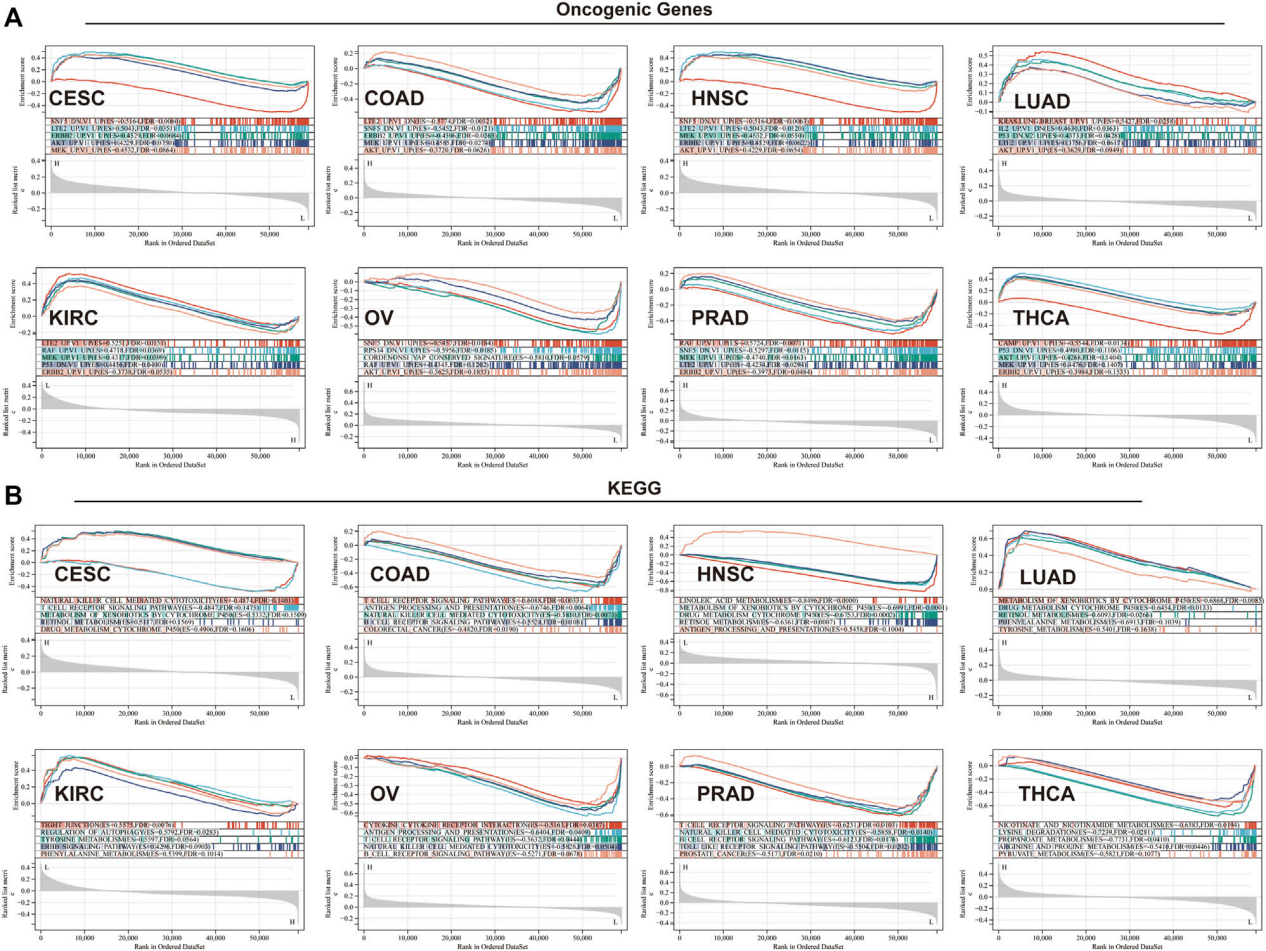
GSEA in eight cancer types. **(A)** Oncogenic functional annotation of TGM3 in eight cancer types. **(B)** KEGG pathway analysis of TGM3 in various cancers. Colored curves indicate significant functions or pathways screened for different tumors. Peaks on the ascending curve (top enrichment) indicate positive association, while peaks on the descending curve (bottom enrichment) indicate negative association.

To elucidate the biological and molecular function of TGM3 in various cancer types, we performed GSEA. Eight cancer types closely related with TGM3 expression were utilized to evaluate the oncogenic functional annotation (Figure 9A) and KEGG pathway analysis (Figure 9B). SNF5, AKT, MEK, ERBB2 and P53 were the most significant oncogenic functional modules in these cancers. In CESC, LUAD, HNSC and KIRC, TGM3 expression was positively correlated with these oncogenic pathways, including AKT-, ERBB2-, LTE2-, and KRAS-related pathways. In contrast, TGM3 expression was negatively correlated with LTE2-, SNF5-, and RAF-related pathways in PRAD, COAD and OV. In THCA, TGM3 expression was negatively correlated with the CAMP oncogenic pathway, while TGM3 expression was positively correlated with the P53, AKT, MEK, and ERBB2 oncogenic pathways. Furthermore, the biological significance of TGM3 expression in the above eight tumors was further explored through KEGG pathway analysis, which detects insignificant but consistent gene sets with differential expression trends as well as determines whether the pathway is activated or inhibited, to identify the top five pathways relevant to certain biochemical function with significant positive or negative association with TGM3 expression in each tumor. The results demonstrated that TGM3 expression was negatively associated with several immune-related pathways, such as natural killer (NK) cell-mediated cytotoxicity, T cell receptor, B cell receptor, and Toll-like receptor signaling pathways in CESC, COAD, PRAD, and OV. Metabolism-related pathways, such as retinol metabolism, xenobiotics and drug metabolism by cytochrome P450, were negatively related with TGM3 expression in HNSC but positively related with TGM3 expression in LUAD. Other metabolism-related pathways, such as nicotinate, lysine degradation, arginine metabolism and pyruvate metabolism, were negatively regulated with TGM3 expression in THCA. In KIRC, TGM3 expression was positively correlated with tight junction, autophagy and the ERBB signaling pathway, which are relevant to tumorigenesis.

## Discussion

TGM3 has been shown to play biological roles in various types of cellular physiological and pathological activities, such as cell differentiation, cell cycle regulation, apoptosis, cytokine/chemokine incretion, immune response regulation and epidermal barrier formation (Liu et al., 2012; Li et al., 2016b; Wu et al., 2018; Feng et al., 2020). Recently, emerging studies have reported various clinical value and physiological function correlations between TGM3 and various cancers, including clinical stage, prognosis, tumor heterogeneity, gene mutation, methylation level, lymphocyte infiltration and immune regulation, etc. Many studies have revealed that the alternation of TGM3 expression level is significantly correlated with cell proliferation, dedifferentiation, migration, invasiveness, high incidence of lymph node metastasis, high incidence rate of recurrence and low survival rate (Smirnov et al., 2019; Hu et al., 2020). However, it remains unknown how TGM3 acts as a cancer-related molecule in the pathogenesis of different tumors. Because no pan-cancer analysis of TGM3 based on databases from various cancers has been reported thus far, we investigated the *TGM3* gene in pan-cancer according to TCGA, TARGET, CCLE and GTEx databases by evaluating its molecular features, genetic alteration, immune infiltration, and relation with clinical characteristics.

Previous studies have suggested a T cell proliferative response toward TGM2 and TGM3 in dermatitis herpetiformis and celiac disease, and they have also demonstrated that the proliferative frequency of antigen-specific T-cell lines (TCLs) and the intensity of T cell proliferation are greatly increased upon continuous stimulation with TGM2 or TGM3 (Lorand and Graham, 2003). It has also been reported that the presence of TGM3/IgA deposits activated the migration of neutrophils to the dermal papilla, which efficiently induces subepidermal differentiation through local skin-produced cytokines and chemokines, such as TNF-α and IL-17A, to cleave the adhesion protein from the basement membrane bands, including collagen VII (Russo et al., 2018). In addition, TGM3 is the crucial autoantigen that recognizes T cells in dermatitis herpetiformis (Hradetzky et al., 2015; Caproni et al., 2021). Another study has revealed that TGM3 expression is higher in atopic dermatitis (AD) patients and is positively linked with disease severity, indicating that TGM3 is an autoallergen immune regulator that actively affected skin inflammation in AD (Su et al., 2020). As an important C-type lectin receptor, DC-specific non-integrin intercellular adhesion molecule (DC-SIGN) specifically recognizes glycosyl structures, thereby mediating DC activation and inflammation *via* the NF-κB signaling pathway, ultimately inducing Th1 polarization. As a protease, TGM3 contains certain glycosyl groups and may be recognized and taken up by DC through the DC-SIGN receptor, thereby mediating downstream immune responses (Trompette et al., 2009). Thus, it is important to elucidate the correlation between TGM3 and immune cell infiltration of different tumors. To evaluate the role of TGM3 related to immune cell infiltration of different types of cancers, a pan-cancer database is a useful and effective tool to preliminarily analyze the infiltration ratio and cell groups with TGM3 alternations in various cancers.

The present findings suggest that TGM3 may be a highly specific diagnostic and prognostic biomarker in different types of carcinomas, especially in epithelial carcinoma. Consistent with our analysis, the expression level of TGM3 is high in hepatocellular carcinoma but low in other epithelial carcinomas, such as laryngeal carcinoma and esophageal carcinoma (Chen et al., 2000; He et al., 2002; Li et al., 2016b; Hu et al., 2020), Kaplan–Meier survival analysis showed that high TGM3 expression signified a poor prognosis for patients suffering from most cancers, except PAAD, which indicated a clear association of TGM3 expression with cancer survival and prognosis, suggesting that TGM3 is a potential biomarker for cancer monitoring. The feasibility of clinical studies and accuracy of results need to be enhanced because OS is not sufficient and death incident caused by non-cancer reasons is not related to tumor biology, invasiveness, or actual response to anti-cancer treatment. Additionally, OS or DSS usually requires a longer follow-up period. Therefore, the use of DFI or PFI in clinical trials may be more effective and objectively reflect the real medical and prognosis condition on patients. Although high TGM3 expression mainly plays a carcinogenic role in most tumor types, the expression level of TGM3 is lower in tumor tissues than normal tissues in some cancers, such as LAML and ALL, and higher TGM3 expression indicates poorer prognosis. Because the online RNA-seq data include bulk-seq data, which contains information of various cells, we focused on comparing paired tumor tissues to shed lighter on the mechanism.

The TME consists of tumor cells, stromal cells (such as fibroblasts), a variety of immune cells (including T lymphocytes, NK cells, macrophages, and DCs), and extracellular matrix (Wei et al., 2021). To further verify the potential relationship between TGM3 and the immune microenvironment, correlation analysis was performed and indicated that the expression of TGM3 in pan-cancer was clearly related to immune checkpoints and regulators in several tumors. The upregulation of checkpoints and regulatory genes may be the result of immune cell activation or altered protein trafficking caused by TGM3 regulation. By elucidating the relationship between TGM3 and immune cell infiltration scores, immune checkpoints, immune activation genes, immunosuppressive genes, chemokines and chemokine receptors, our findings provided insights into the functional role of TGM3 in pan-cancer and highlighted potential mechanisms by which TGM3 affects the TME and cancer immunotherapy.

Tumor immune cell infiltration refers to the movement of immune cells from the blood into the tumor tissue to exert their effects. The infiltration of immune cells in tumors is closely related to clinical outcome, and immune cell infiltration in tumors may serve as a drug target to improve patient survival. (Di Caro et al., 2013). Additional findings have illustrated the association of tumor progression with different immune cell infiltration results from host immunosurveillance deficiency and the escape of cancer cells (Spinner et al., 2019). Therefore, it is essential to clarify the underlying mechanism of infiltrating immune cells involving TGM3 in the TME. Cytotoxic CD8+ T cells and their activated cytotoxic T cells have a functional impact on cellular immunity, efficiently eliminating malignant cells in tumor tissues and providing long-term protective immunity, whereas CD4+ T cells assist the activation and memory of cytotoxic CD8+ T cells, regulating antigen-presenting cells (APCs) to provide stronger antigenic signals to naive CD8+ T cells (Nakanishi et al., 2009; Ma et al., 2021; Pipkin, 2021). In the present study, we showed that TGM3 expression was negatively correlated with B cells, DCs and neutrophil cells in most cancers, including STAD, TGCT, PRAD, LUSC, THYM and HNSC, according to the TIMER database. These findings helped to explain the tumorigenic role of TGM3 through cellular immunity dysregulation in most tumor types. For example, Tregs help malignant tumor cells escape attack from cytotoxic CD8+ T cells due to their suppressive function (Liu et al., 2016; Togashi et al., 2019). In the present study, however, Tregs were not significantly altered according to the analysis of immune cell infiltration using the CIBERSOFT database (Supplementary Figure S5). TG, the autoantigen involved in celiac illness, deamidates gluten peptides to enhance the affinity to HLA-DQ2 and/or HLA-DQ8, thereby strongly activating CD4+ T helper cells, ultimately leading to mucosa inflammation (Sardy et al., 2002; Antiga et al., 2015; Antiga et al., 2019). In the present study, CD4+ cells, neutrophils, and DCs were also significantly altered in many cancers with the change of TGM3 expression level. However, additional experiments are required to elucidate the relation and mechanism of TGM3 immune regulation in diverse tumors.

Cancers arise due to gene mutations, including both germline and somatic mutation to some extent, providing a selective growth and metastasis superiority in cancer cells (Vogelstein and Kinzler, 2004; Greenman et al., 2007). In the present study, we assessed the patterns of TGM3 mutation in pan-cancer. Several studies have found loss or impairment of enzymatic activity in inherited skin diseases caused by TGM3 mutations, which can be compensated by TGMs or replaced by non-TGM3 protein functions (Scherer, 1985). Therefore, the role of TGM3 gene alteration and mutation as a tumor marker should be further investigated. The epigenetics of a normal cell can be compared to DNA hypomethylation in tumor cells to explain the role of methylation and the inactivation of tumor suppression genes in cancer, and epigenetics also play a role in cancer management (Esteller, 2008). DNMT1, DNMT2, DNMT3A and DNMT3B are involved in the production and maintenance of DNA methylation (Lyko, 2018). The relation between TGM3 methylation levels and DNMTs in different tumors has not been previously reported. Detection of abnormalities in gene CpG island methylation patterns has been suggested as a novel approach to predict cancer development (Schinke et al., 2010). Therefore, the detection of TGM3 methylation may shed light on the exploration of potential tumor molecular biomarkers.

Current methods for detecting TMB refer to the number of somatic mutations, including point mutations and indels, per megabase (Mb) of patient-targeted sequencing coding regions. Non-synonymous mutations in somatic cells can be manifested as changes in RNA and protein levels, and the generated neoantigens (or neoepitopes) and protein fragments/peptides, which are recognized by the autoimmune system as non-self antigens, activate T cells and cause immune response (Campbell et al., 2017). Widespread use of monoclonal antibodies for immunotherapy increases the burden of mutation and treatment resistance (Chan et al., 2015; Rizvi et al., 2015; Chan et al., 2019). MSI is a potential predictive marker for immunotherapy to ensure high clinical effects of tumor treatment due to its immunogenic and extensive T cell infiltration (Cortes-Ciriano et al., 2017). In the present study, TGM3 was positively correlated with TMB and MSI in SARC, which indicated that TGM3 may have a direct effect on the immunotherapeutic response of SARC. Interestingly, TGM3 positively regulated TMB but negatively regulated MSI in KIPAN, requiring further investigation to explore whether KIPAN with high expression of TGM3 is sensitive to PD-1/PD-L1 treatment.

At present, ICI therapy has become the focus of cancer treatment. This type of therapy aims to help the immune system recognize and attack tumor cells. The main target molecules are programmed death receptor ligand 1 (PD-L1), programmed death receptor 1 (PD1) and cytotoxic T lymphocyte associated protein 4 (CTLA4). Recent studies have revealed two different mechanisms of tumor immune escape: in some tumors, although cytotoxic T cells are highly infiltrated, these T cells are often in a dysfunctional state; in other tumors, immunosuppressive factors can remove T cells infiltrating tumor tissue. Therefore, a new computing architecture -TIDE score is used to integrate the two mechanisms of tumor immune escape (Jiang et al., 2018). A high TIDE score indicates that poor efficacy and short survival period after ICI therapy. Here we regarded TGM3 as a new regulator of ICI drug resistance. It was found low expression level of TGM3 in patients with better response to immunosuppressive therapy at the immune checkpoint in KICH, KIRC, KIRP, CHOL and THCA. While in the clinical patients of BRCA, ESCA, HNSC, LAML, OV, PRAD and UCEC, the expression level of TGM3 in patients with effective therapy was always higher than that in patients with ineffective ICI therapy.

Our enrichment analyses suggested that TGM3 may potentially affect the etiology or pathogenesis of cancer by associating with several classic oncogenic signature gene-related pathways and functioning in cell junction, B/T cell activation, immune response, immune regulation, signaling and metabolic pathways. In the present study, we selected eight cancer types, some of which belonging to epithelial neoplasms and others with significant changes in TGM3 expression levels, for analysis. The data were consistent with classic tumorigenesis genes and pathways of these cancers, indicating that expression of TGM3 on tumor cells may have be regulated by B cell and T cell activation linker proteins, thereby affecting the immune reaction, and promoting both immune and oncogenic pathways. GSEA based on KEGG and oncogenic functional annotation, indicated that TGM3 regulated multiple important oncogenic functional modules in tumors, and that TGM3 protein or transcripts was involved in the regulation of potential cancer immunogenicity and immunotherapeutic effects in various cancers. We also investigated whether TGM3 leading to activation or inhibition of this pathway is closely related to tumor heterogeneity. In tumor immunology pathways, TGM3 was negatively correlated with a variety of immune-related pathways, such as NK cell-mediated cytotoxicity, T cell receptors, B cell receptors and toll-like receptor signaling pathways, in CESC, COAD, PRAD and OV. These findings prompted us to consider the role of TGM3 in cancer immunology. Additionally, several classic metabolism-related pathways concerned with tumorigenesis or therapeutic target were selected. Higher serum retinol levels are associated with lower overall mortality, including risk of death from cardiovascular, cardiac, and respiratory diseases (Huang et al., 2021), and CYP450 gene polymorphisms are associated with tumor susceptibility (Ruwali and Parmar, 2010). The metabolism of certain amino acids within the TME may be restricted, including arginine, tryptophan, alanine, serine and glycine, which are required not only for tumor cell proliferation but also for the maintenance of cytotoxic T lymphocyes (CTL) function. In addition to its cell-autonomous role as a survival mechanism for nutrient-starved tumor cells, autophagy controls how tumor cells increase TME-localized immunosuppression as well as systemic organism-dependent immunosuppressive mechanisms. In addition to nutrient competition, there is also metabolic interference between different cell types in the TME, a strategy by which tumor cells continue to grow under adverse conditions (Martinez-Reyes and Chandel, 2021). Hence metabolic enzymes are attractive potential therapeutic targets.

To the best of our knowledge, this is the first study to conduct a comprehensive analysis of TGM3 in pan-cancer. The present study shed light on the importance of TGM3 as a cancer prognostic biomarker and effective predictor of immunotherapy response as well as the relationship of TGM3 with immune modulators, prognosis, immune regulation and TME, aiding in the understanding of the potential mechanism between TGM3 and tumorigenesis and development. The present results were based on bioinformatics analysis and no experiments were performed to verify these results. Thus, we intend to perform further research to verify the correlation between TGM3 and carcinogenesis.

## Data Availability

The original contributions presented in the study are included in the article/Supplementary Material, further inquiries can be directed to the corresponding authors.
