# Supplementary material for: Clinical and immunological characteristics of TGM3 in pan-cancer: A potential prognostic biomarker
Source: Front Genet. 2023 Jan 6;13:993438. doi: 10.3389/fgene.2022.993438 (PMC9852731; doi:10.3389/fgene.2022.993438)
Supplement: Supplementary file 1 [file Presentation1.pdf]

Supplemental Material

	Samples		Risk Ratio			Statistical Test					
	Treated	Reference	HR	95%CI		Likelihood ratio test		Wald test		logrank test	
				Lower	Upper	W value	p value	W value	p value	W value	p value
KIPAN	H	L	1.73	1.33	2.25	16.42	5.10E-05	16.72	4.30E-05	17.13	3.50E-05
KIRC	H	L	1.78	1.3	2.45	12.53	4.00E-04	12.68	3.70E-04	13.03	3.10E-04
SKCM	H	L	1.41	1.07	1.87	5.98	0.01	5.82	0.02	5.87	0.02
LAML-TCGA	H	L	2.16	1.49	3.12	15.33	9.00E-05	16.75	4.30E-05	17.55	2.80E-05
ALL	H	L	2.47	1.5	4.07	11.63	6.50E-04	12.54	4.00E-04	13.37	2.60E-04
UCS	H	L	2.41	1.2	4.81	5.73	0.02	6.15	0.01	6.54	0.01
LAML-TARGET	H	L	2.05	1.24	3.37	8.4	3.80E-03	7.89	5.00E-03	8.23	4.10E-03
PAAD	H	L	0.54	0.33	0.89	6.45	0.01	5.82	0.02	5.99	0.01

Table S1. Details of Kaplan–Meier OS of TGM3 in indicated tumor types from TCGA and TARGET databases with significant P-value.

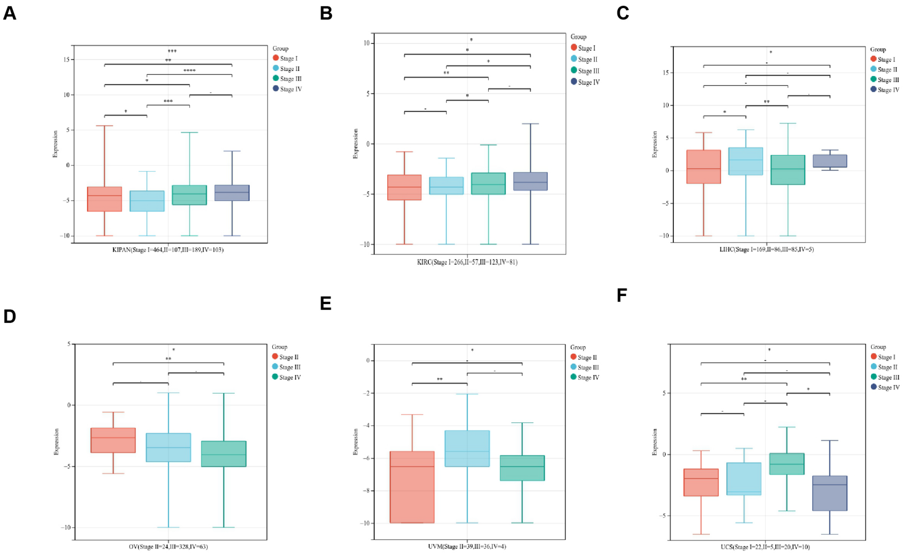

Label	Experiment group (Mean±std)	Control Group (Mean±std)	T test	P value
KIPAN (Stage I=464,II=107,III=189,IV=103)	Stage I(-4.70±2.67)	Stage III(-4.24±2.41)	0.03	3.00E-04
	Stage I(-4.70±2.67)	Stage II(-5.37±2.51)	0.01	
	Stage I(-4.70±2.67)	Stage IV(-4.07±2.07)	9.40E-03	
	Stage III(-4.24±2.41)	Stage II(-5.37±2.51)	2.10E-04	
	Stage III(-4.24±2.41)	Stage IV(-4.07±2.07)	0.53	
	Stage II(-5.37±2.51)	Stage IV(-4.07±2.07)	6.10E-05	
KIRC (Stage I=266,II=57,III=123,IV=81)	Stage III(-4.14±1.95)	Stage II(-4.85±2.32)	0.05	0.01
	Stage III(-4.14±1.95)	Stage IV(-4.08±2.10)	0.84	
	Stage III(-4.14±1.95)	Stage I(-4.74±2.29)	7.30E-03	
	Stage II(-4.85±2.32)	Stage IV(-4.08±2.10)	0.05	
	Stage II(-4.85±2.32)	Stage I(-4.74±2.29)	0.75	
	Stage IV(-4.08±2.10)	Stage I(-4.74±2.29)	0.02	
LIHC (Stage I=169,II=86,III=85,IV=5)	Stage I(0.35±3.07)	Stage II(1.27±3.01)	0.02	0.04
	Stage I(0.35±3.07)	Stage III(-7.2e-4±3.31)	0.41	
	Stage I(0.35±3.07)	Stage IV(1.35±1.35)	0.18	
	Stage II(1.27±3.01)	Stage III(-7.2e-4±3.31)	9.50E-03	
	Stage II(1.27±3.01)	Stage IV(1.35±1.35)	0.91	
	Stage III(-7.2e-4±3.31)	Stage IV(1.35±1.35)	0.09	
OV (Stage II=24,III=328,IV=63)	Stage IV(-4.19±2.49)	Stage III(-3.55±2.11)	0.06	0.03
	Stage IV(-4.19±2.49)	Stage II(-2.97±1.49)	6.60E-03	
	Stage III(-3.55±2.11)	Stage II(-2.97±1.49)	0.08	
UVM (Stage II=39,III=36,IV=4)	Stage III(-5.64±2.07)	Stage II(-7.12±2.31)	4.40E-03	0.02
	Stage III(-5.64±2.07)	Stage IV(-6.70±2.52)	0.47	
	Stage II(-7.12±2.31)	Stage IV(-6.70±2.52)	0.77	
UCS (Stage I=22,II=5,III=20,IV=10)	Stage II(-2.42±2.38)	Stage III(-0.81±1.72)	0.21	0.02
	Stage II(-2.42±2.38)	Stage I(-2.32±1.75)	0.93	
	Stage II(-2.42±2.38)	Stage IV(-2.77±2.29)	0.8	
	Stage III(-0.81±1.72)	Stage I(-2.32±1.75)	7.40E-03	
	Stage III(-0.81±1.72)	Stage IV(-2.77±2.29)	0.03	
	Stage I(-2.32±1.75)	Stage IV(-2.77±2.29)	0.59	

Figure S1 and Table S2. Pan-cancer TGM3 expression in different clinical stages. (A–F) Pan-cancer differential expression of TGM3 in clinical stages in indicated tumor types from TCGA database. *P<0.05, **P<0.01, ***P<0.001, and ****P<0.001; -, not significant.

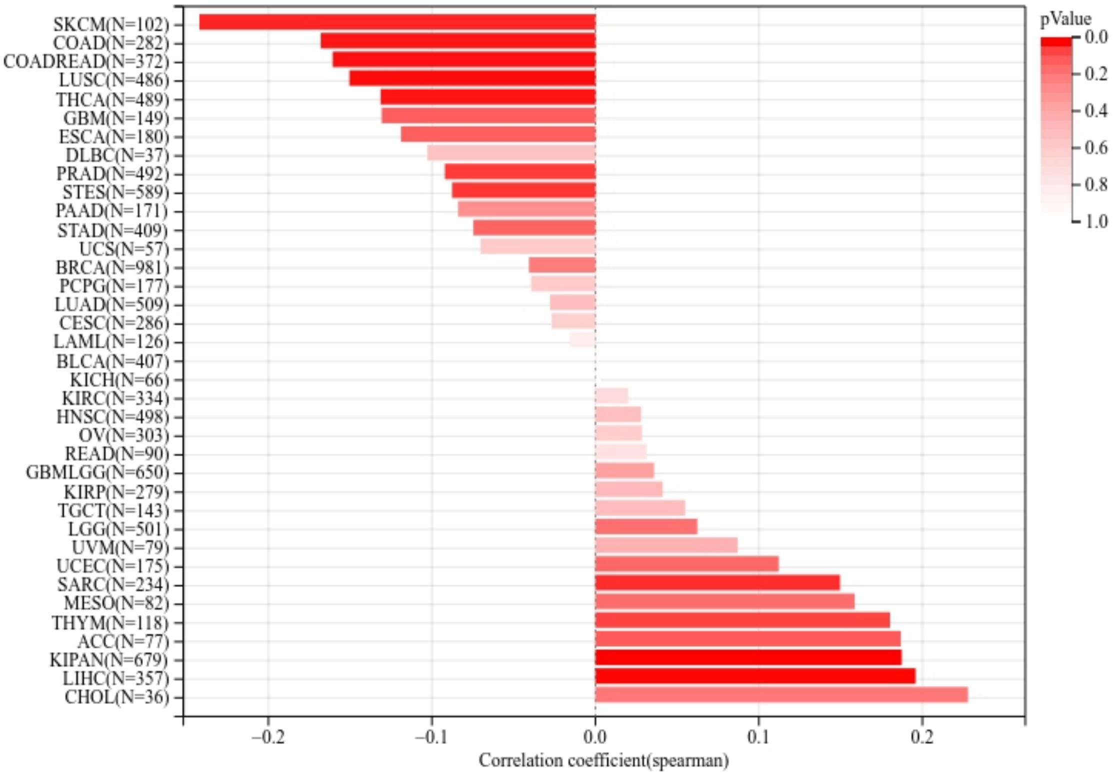

Figure S2. Relation between TMB and TGM3 mRNA expression levels in various tumors. TMB was counted by total mutation incidences per million base pairs in each tumor.

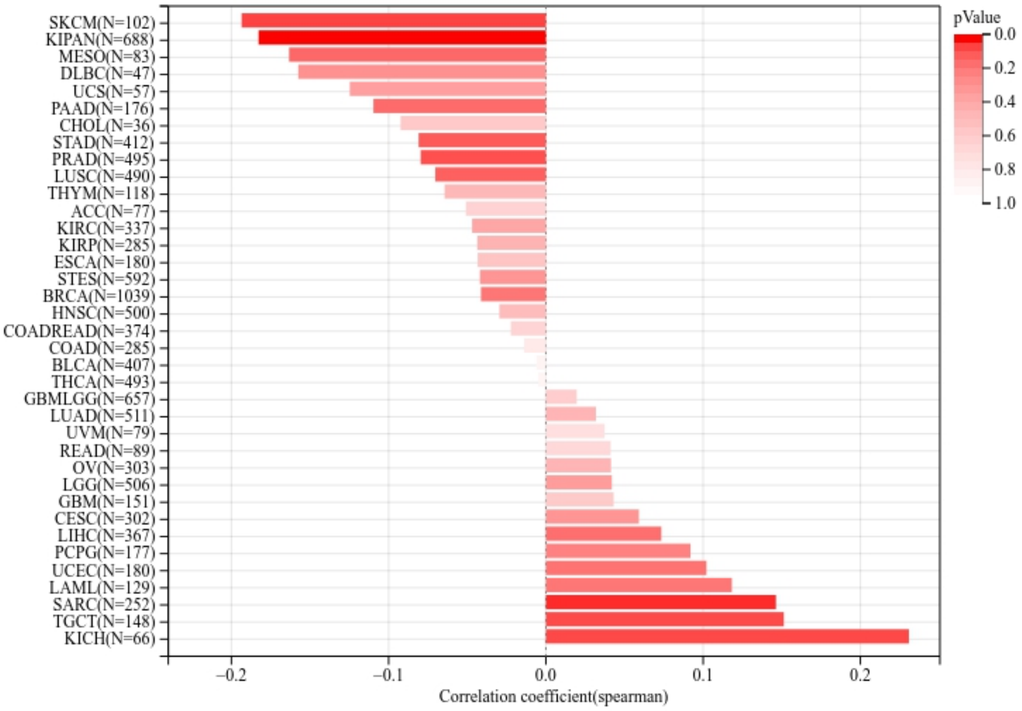

Figure S3. Relation between MSI and TGM3 mRNA expression levels in various tumors. MSI was counted by total incidence of deletion or insertion in repeating sequences per million base pairs.

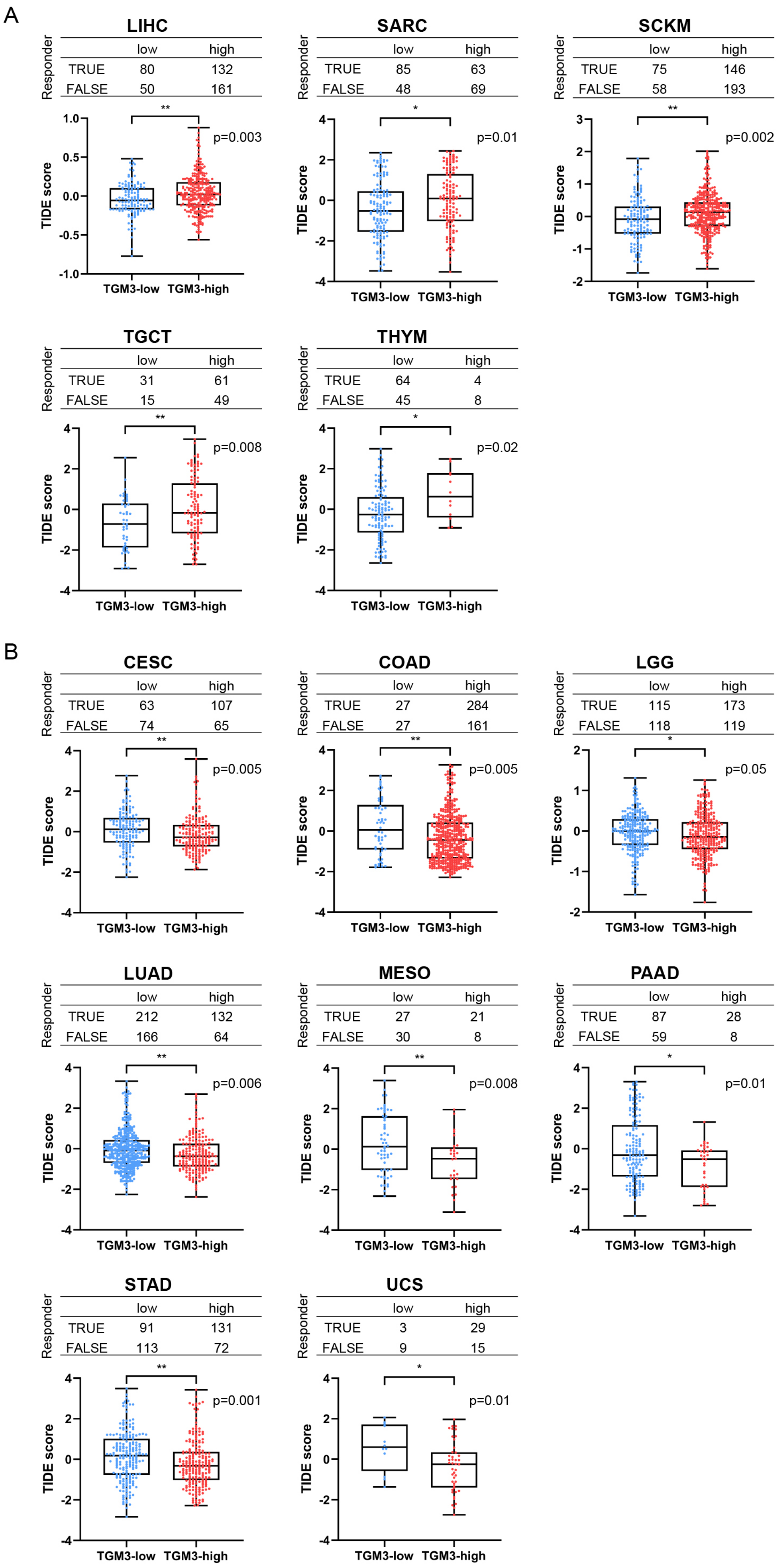
A
LIHC
Responder
low high
TRUE 80 132
FALSE 50 161
**
p=0.003
TIDE score
TGM3-low TGM3-high
SARC
Responder
low high
TRUE 85 63
FALSE 48 69
*
p=0.01
TIDE score
TGM3-low TGM3-high
SCKM
Responder
low high
TRUE 75 146
FALSE 58 193
**
p=0.002
TIDE score
TGM3-low TGM3-high
TGCT
Responder
low high
TRUE 31 61
FALSE 15 49
**
p=0.008
TIDE score
TGM3-low TGM3-high
THYM
Responder
low high
TRUE 64 4
FALSE 45 8
*
p=0.02
TIDE score
TGM3-low TGM3-high
B
CESC
Responder
low high
TRUE 63 107
FALSE 74 65
**
p=0.005
TIDE score
TGM3-low TGM3-high
COAD
Responder
low high
TRUE 27 284
FALSE 27 161
**
p=0.005
TIDE score
TGM3-low TGM3-high
LGG
Responder
low high
TRUE 115 173
FALSE 118 119
*
p=0.05
TIDE score
TGM3-low TGM3-high
LUAD
Responder
low high
TRUE 212 132
FALSE 166 64
**
p=0.006
TIDE score
TGM3-low TGM3-high
MESO
Responder
low high
TRUE 27 21
FALSE 30 8
**
p=0.008
TIDE score
TGM3-low TGM3-high
PAAD
Responder
low high
TRUE 87 28
FALSE 59 8
*
p=0.01
TIDE score
TGM3-low TGM3-high
STAD
Responder
low high
TRUE 91 131
FALSE 113 72
**
p=0.001
TIDE score
TGM3-low TGM3-high
UCS
Responder
low high
TRUE 3 29
FALSE 9 15
*
p=0.01
TIDE score
TGM3-low TGM3-high

Figure S4. The relationship between TGM3 and patient response to ICI therapy in other cancer types with statistical significance. (A) Low expression of TGM3 group had a better therapeutic ICI response in LIHC, SARC, SKCM, TGCT, and THYM. (B) High TGM3 expression group had a better therapeutic ICI response in CESC, COAD, LGG, LUAD, MESO, PAAD, STAD, and UCS.

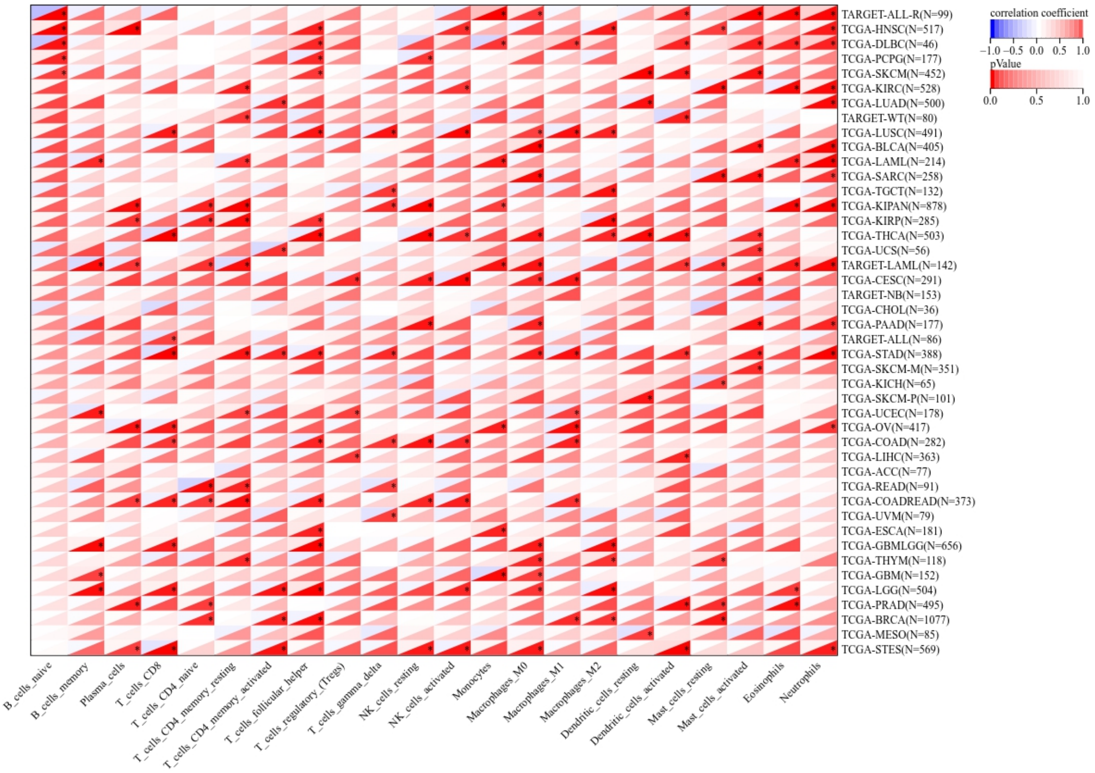

Figure S5. Correlation between TGM3 expression and tumor-infiltrating immune cells as calculated by CIBERSOFT database. The upper triangle in each tile indicates coefficients calculated by Spearman's correlation test, and the lower triangle indicates the P-value. *P<0.05
